# Flexible endoscopy in the visualization of 3D-printed maxillary sinus and clinical application

**DOI:** 10.1007/s00464-022-09410-8

**Published:** 2022-07-26

**Authors:** ZhengRong Xu, Xin Zhang, Xin Dou, ChuanYao Lin, HanDong Wang, ShengHua Song, ChenJie Yu, XinYan Cui, Xia Gao

**Affiliations:** 1 Department of Otolaryngology Head and Neck Surgery, Jiangsu Provincial Key Medical Discipline (Laboratory), Nanjing Drum Tower Hospital, The Affiliated Hospital of Nanjing University Medical School, Nanjing, China; 2 Department of Rheumatology and Immunology, Nanjing Drum Tower Hospital, The Affiliated Hospital of Nanjing University Medical School, Nanjing, China; 3 Research Institute of Otolaryngology, Nanjing Drum Tower Hospital, The Affiliated Hospital of Nanjing University Medical School, Nanjing, China; 4 Department of Otorhinolaryngology, The First Affiliated Hospital, Nanjing Medical University, Nanjing, China; 5 Department of Radiology, Nanjing Drum Tower Hospital, The Affiliated Hospital of Nanjing University Medical School, Nanjing, China

**Keywords:** Flexible endoscopy, Maxillary sinus, 3D print, Postoperative follow-up

## Abstract

**Background:**

During postoperative follow-up, the visible range of maxillary sinus (MS) is limited, even combining 0° and 70° rigid endoscopes together. Flexible endoscope has been used in larynx examinations for a long time, but rarely in nasal cavity and sinus. We aimed to evaluate the application values of rigid and flexible endoscopes for visualization of MS.

**Methods:**

We followed up 70 patients with lesions in MS via both rigid and flexible endoscopes. In addition, we used thin-slice CT image of the sinus to create a MS model and divided it into two parts for 3D printing. The inner surface of the 3D-printed sinus was marked with grid papers of the same size (5 mm × 5 mm), then the visual range under rigid endoscopes with different angle and flexible endoscopes was calculated and analyzed.

**Results:**

In clinical follow-up, we found that flexible endoscopy can reach where rigid endoscopy cannot, which is more sensitive than medical imaging. Endoscopes showed the largest observation range of the posterolateral wall, more than half of which can be visualized by 0° endoscope. Almost all of the posterolateral wall can be revealed under 45° endoscope, 70° endoscope and flexible endoscope. The visual range of each wall under flexible endoscope is generally greater than that under rigid endoscopes, especially of the anterior wall, medial wall and inferior wall.

**Conclusion:**

There was obviously overall advantage of using flexible endoscope in postoperative follow-up of MS lesions. Flexible endoscopy can expand the range of observation, and improve the early detection of the recurrent lesion. We recommend flexible endoscope as a routine application.

**Graphical abstract:**

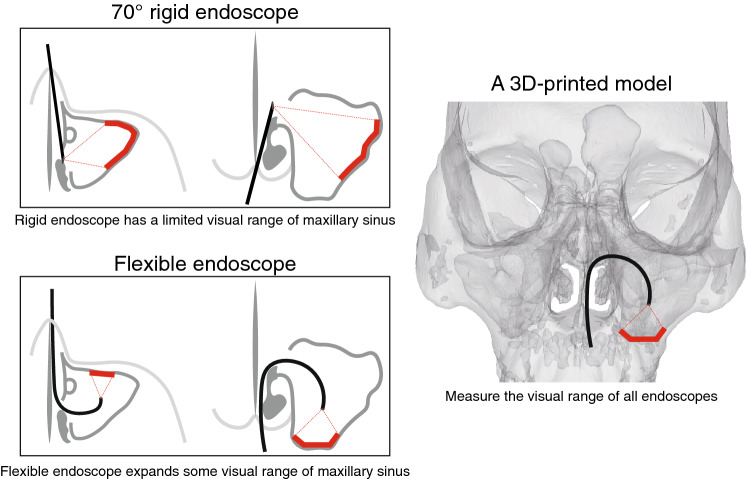

**Supplementary Information:**

The online version contains supplementary material available at 10.1007/s00464-022-09410-8.

The maxillary sinus (MS) is the largest sinus in human body, with an average volume of 12–20 ml [1, 2]. In general, the pyramid-shaped MS consists of five walls, including inferior wall, superior wall, anterior wall, medial wall, and posterolateral wall [3]. There are many anatomical variations of MS, such as hypoplasia, antral septa, or bone exostosis [4].

Endoscopy is widely used in the examination of nasal cavity and nasopharynx. Traditional external approach has been replaced by endoscopic intranasal approach in surgery [5]. For MS surgery, the scope is limited when we remove the lesion through the natural ostium. Though lesions can be completely removed through other unnatural approaches, such as prelacrimal recess and canine fossa [6–9], these extra pathways are often closed after surgery. Therefore, the postoperative endoscopic follow-up of MS disease largely depends on the natural ostium.

For benign and malignant lesions originating from MS, observation of the original site is very important in follow-up. In particular, the close follow-up and early detection of inverted papilloma and malignant tumors are crucial for the prognosis [10–13]. For postoperative follow-up of MS diseases, CT and MRI have limited values for detecting small neoplasms. It is difficult to diagnose due to the postoperative mucosal edema and inflammation, resulting in a large number of false positives [12]. Therefore, radiography is much less intuitive and reliable than endoscopy here. In addition, the radiation risk of CT and the economic cost of MRI make them difficult to be used as routine follow-up techniques. However, it’s hard for endoscopes to reveal the full view of inner surfaces of MS due to its complicated anatomy, especially for conventional 0° rigid endoscope. Although endoscopes with other angles are widely used in MS surgery, they’re still not routine devices for follow-up. Flexible endoscopy is a routine practice of otolaryngology for viewing nasal cavity, nasopharynx and larynx while is rarely used in paranasal sinus. Although some literature reported the usage of flexible endoscope in sinus lesions, more specific research data are needed [9, 14–16].

Three-dimensional (3D) printing technology has developed rapidly and been widely used in medical field in recent years [17–19]. At present, computer modeling based on CT, MRI or ultrasound scan data, combined with 3D printing technology, can be used to restore individualized anatomical structures consistent with the actual disease [20]. It helps surgeons to view and understand the anatomical structure of the disease before surgery, and make accurate surgical intervention program [21]. At the same time, individualized and customized implants can be formulated according to the patient's anatomical conditions [22, 23]. Since bone is the easiest biological tissue to be replicated [24], 3D printing is widely used in the fields of orthopedic surgery, dentistry, maxillofacial surgery and plastic surgery [25–27]. In otolaryngology, 3D printing has been used in preoperative planning, education, prosthesis, transplantation and reconstruction [28]. The accuracy of the application in the MS has also been confirmed [29].

In this study, we followed up patients after maxillary sinus surgery with flexible endoscope, which is very valuable for lesions in specific area. Therefore, we wanted to study the advantages of flexible endoscope in this regard. However, due to the difficulty of actual clinical measurement, we designed a 3D-printed MS model, whose inner surface was covered with grid papers. The visual range in the model under flexible and rigid endoscopes was calculated and analyzed. Our study provides a theoretical basis for the selection of endoscopes in the follow-up of MS surgery.

## Materials and methods

This study included the retrospective data of preoperative and postoperative follow-up examinations of two patients and one volunteer. It was considered exempt by the ethics review committee at the Nanjing Drum Tower Hospital. Informed consents have been obtained from patients in this study for scientific research.

### Cleaning and sterilizing endoscopes

Rigid endoscopes including 0°, 45°, and 70° endoscopes (KARL STORZ, Germany), and flexible endoscope (ENF-VH, Olympus, Japan) were cleaned in advance before operation. The endoscopes were scrubbed with 3 M endoscope special all-around powerful cleaning solution, disinfected with 0.55% ortho-phthalaldehyde, and dried with 75–95% ethanol. This method refers to the health industry standard of the People's Republic of China, "Regulation for cleaning and disinfection technique of flexible endoscope”, WS 507-2016, which was published on June 1, 2017.

### Models

**Fig. 1:**
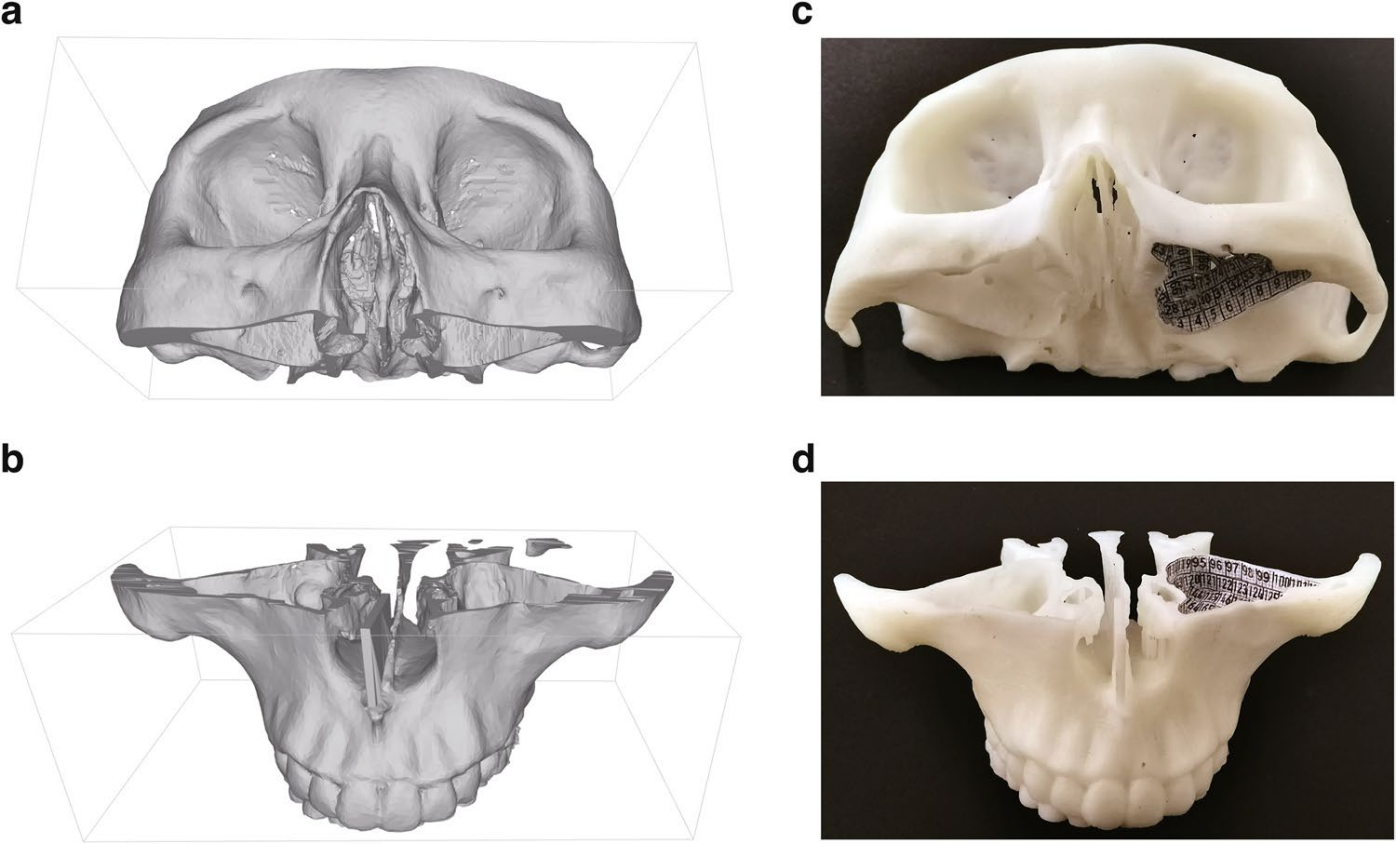
The 3D-printed model of MS. **a**, **b** The bone structure was reconstructed by CT image of paranasal sinus from a healthy volunteer. It was horizontally divided into upper (**a**) and lower (**b**) parts from the plane below the natural ostium of MS. **c**, **d** 3D-printed model was used in the following endoscopy. Marked grid papers were stuck to the inner surface of the left MS

A volunteer with healthy paranasal sinuses was scanned by Computed Tomography scanner (GE Revolution maxima, American). The DICOM file of thin-slice image (0.63 mm reconstruction increment) was imported into E-3d Master Edition Modeling and Design Software (V17.08) (Central South E3D Digital Medical and Virtual Reality Research Center, China). The MS model was built by separating the target area through the "intelligent segmentation" function. The model was then divided horizontally below the plane of natural ostium of MS into two parts, optimized by using the "simple segmentation" function (Fig. 1a, b). Next, the model was printed by the stereolithography 3D printer (Zhongrui Technology iSLA880 light-curing laser 3D printer, China) using the material of photosensitive resin.

Some 5 × 5mm^2^ gride papers marked with different numbers were introduced inside the sinus pressing against all the walls (Fig. 1c, d). Next, two parts of the model were stuck together. Three rigid endoscopes and flexible endoscope were used to survey the walls of MS. The marked numbers observed on each wall under different endoscopes were recorded. In order to simplify the calculation, we simply regarded the visual area of a grid paper that can’t be fully displayed as half of the total grid paper. Finally, the visual range map was built and the range was calculated and analyzed.

### Patients

Patients underwent successful surgical ablation of inverted papilloma or tumor in MS through the prelacrimal recess approach. After removal of the lesion, the incision in medial wall of MS was sewed up. In order to simplify the follow-up, the MS natural ostium was enlarged from the osseous nasolacrimal duct in anterior, to the posterolateral wall of MS in posterior. During the postoperative follow-up process, MRI and CT scan were performed, and both rigid and flexible endoscopes are introduced to record and compare the visual range.

## Results

In clinical application, the follow-up of patients after maxillary sinus surgery is often difficult. CT and MRI examinations are not as intuitive as endoscopy, while it is difficult to observe certain area with rigid endoscopes. We innovatively use flexible endoscope to observe lesions in MS. In a total of 70 followed-up patients, two typical and interesting cases are put forth below to evaluate the application value of different endoscopes.

### Case1

In a patient with inverted papilloma of the MS, a bone septum arising from the floor of the MS can be bypassed using flexible endoscope, revealing the anterior compartment of alveolar recess.

**Fig. 2:**
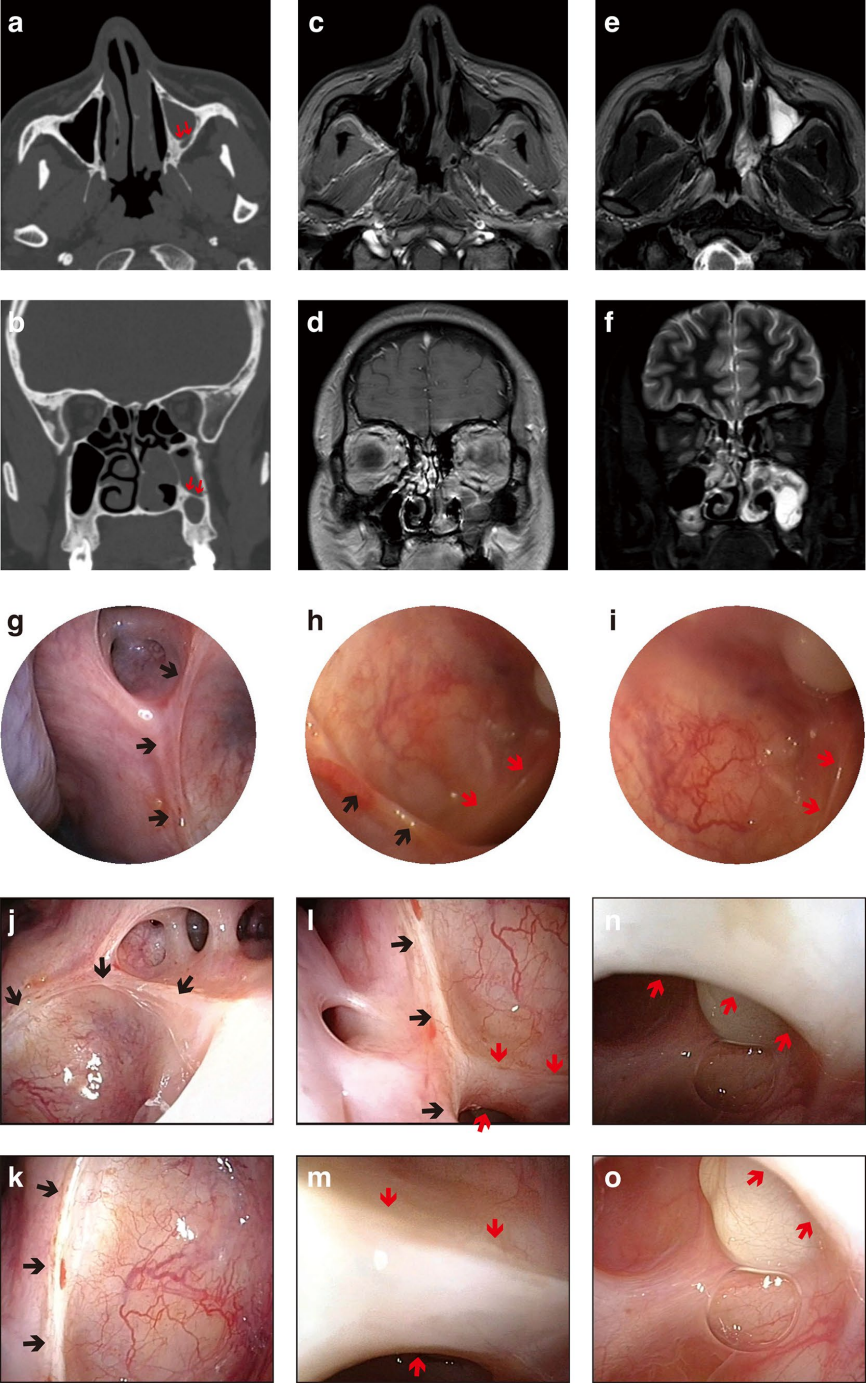
Bone septum on the inferior wall of MS bypassed via flexible endoscope in Case 1. **A–f** Preoperative medical images of the patient, including CT images (**a**, **b**), T1 weighted contrast-enhanced MRI (**c**, **d**) and T2 weighted MRI (**e**, **f**). A bone septum (red arrows) was revealed clearly on CT image. **g–i** Inner surfaces of MS were observed by endoscopes through ostium (black arrows). Postoperative follow-up of MS using 0° (**g**) and 70° (**h**, **i**) rigid endoscopes. **j–o** The bone septum (red arrows) could be bypassed using flexible endoscope (Color figure online)

The preoperative CT image showed lesions in the left MS, which has a bone septum on the inferior wall (Fig. 2a, b). The lesions with secretion retention could be enhanced on MRI scans (Fig. 2c–f). The inverted papilloma, confirmed by postoperative pathology, arises from the junction of the medial and inferior wall. The tumor was resected completely along with edema polyp in the alveolar crypt through prelacrimal recess approach. After the suspicious bones were removed, the mucosa at the anterior of inferior turbinate was sewed up. In postoperative follow-up, the ostium, posterolateral wall and part of the superior wall of the MS could be observed with 0° rigid endoscope, but the inferior wall couldn’t be seen (Fig. 2g). The visual range under 70° endoscope could include the posterior compartment of alveolar recess, and just reach the bone septum in the floor of the MS (Fig. 2h, i). In contrast, the bone septum could be bypassed by applying flexible endoscope, visualizing the anterior compartment of alveolar recess, where a small cyst-like lesion exists (Fig. 2j–o).

### Case 2

In a patient with malignant tumor of the MS, suspicious lesions at the original site of tumor could be detected by flexible endoscopy, preventing recurrence in early stage.

**Fig. 3:**
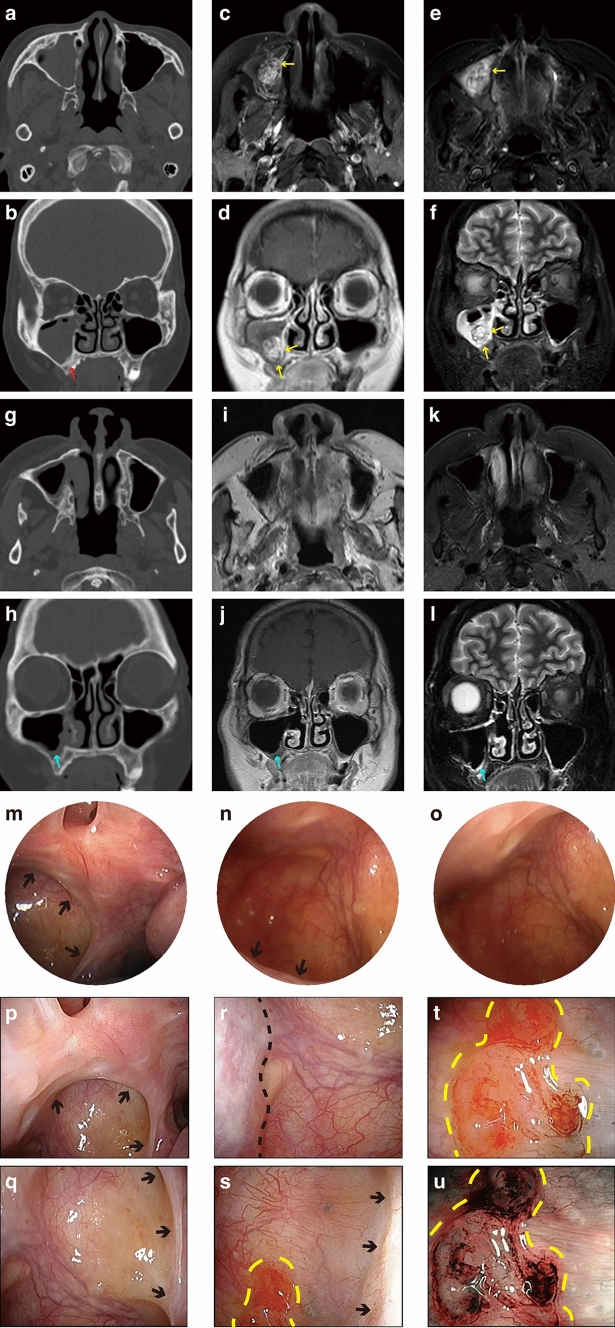
Lesion on the floor of MS detected by flexible endoscope in Case 2. **a–f** Preoperative medical images of the patient, including CT images (**a**, **b**), T1 weighted contrast-enhanced MRI (**c**, **d**) and T2 weighted MRI (**e**, **f**). The right MS with bone cortical discontinuity (red arrow) of the inferior wall was filled with lesions. The tumor located mainly at the medial and inferior wall (yellow arrows). **g–l** Images between two surgeries, including CT images (**g**, **h**), T1 weighted contrast-enhanced MRI (**i**, **j**) and T2 weighted MRI (**k**, **l**). The MS exhibited postoperative signals, suggesting thickening of the sinonasal mucosa and fibrotic scar probably (blue arrow). **m–o** Views of MS were observed by 0° (**m**) and 70° (**n–o**) endoscopes before second surgery. The lesion could hardly be found under 70° endoscope. **p–u** Views of MS were observed by flexible endoscope before second surgery. The anterior wall was presented (black dotted line). After entering the ostium (black arrows) of MS, the whole lesion (yellow dotted line) could be seen. The lesion was vascular dilatation under NBI module (**u**) (Color figure online)

The right MS with bone cortical discontinuity of the inferior wall was filled with lesions, which were significantly enhanced on MRI (Fig. 3a–f). During the first operation, the original site of the tumor was observed mainly at the medial wall and the inferior wall, consistent with imaging characteristics (Fig. 3c–f). The tumor with the affected bone was completely removed via prelacrimal recess approach. Postoperative pathology revealed sinonasal low-grade non-intestinal-type adenocarcinoma.

One year after the first surgery, recurring blood-stained nasal discharge were reported. However, the MS exhibited postoperative signals, suggesting thickening of the sinonasal mucosa and fibrotic scar probably, without obvious new lesions (Fig. 3g–l). We further performed endoscopy to evaluate the disease. The mucosa is smooth and no lesions could be revealed under 0° endoscope (Fig. 3m) or 70° endoscope (Fig. 3n, o). The visible range could be expanded using flexible endoscope, revealing the total suspicious lesion located on the floor, the anterior wall and part of the medial wall of MS (Fig. 3p–u, Supplementary Video). In addition, the narrow band imaging (NBI) module of the flexible endoscope was performed for characterizing the lesion’s features, including vascular dilatation, mucosal edema, and hyperplastic granulation tissue. No characteristic abnormal vascular pattern was observed under NBI-endoscopic evaluation (Fig. 3u). The patient underwent lesion resection through prelacrimal recess again, and the postoperative pathology was foreign body granuloma. There was no further bleeding after surgery.

In these two clinical cases, we preliminarily confirmed the advantage of flexible endoscopy. However, it is difficult to measure the specific visual range in human body. Comparing the visual range of rigid and flexible endoscope, we built a 3D-printed MS model. The grid papers marked with different numbers were used to cover the entire bony inner walls of the left MS of 3D-printed model (Supplementary Table 1). The total internal surface area of the MS counted by 207 pieces of papers is about 51.75 cm^2^ (Table 1). The counts of grid papers on each wall vary, indicating their difference in area. Among, the area of inferior wall is the smallest, with 23 grid papers covered, equivalent to 5.75 cm^2^ (Table 1). The posterolateral wall, with the largest area, provides a total area of 13.5 cm^2^ marked with 54 grid papers (Table 1). The other three walls, including superior wall, anterior wall, and medial wall, are 8.75 cm^2^, 10.75 cm^2^, and 13cm^2^, respectively (Table 1).

**Table 1:** Statistics of counts and area of walls in MS

The wall of MS	Total counts	Area (cm^2^)
Anterior wall	43	10.75
Posterolateral wall	54	13.5
Medial wall	52	13
Superior wall	35	8.75
Inferior wall	23	5.75
Total	207	51.75

**Fig. 4:**
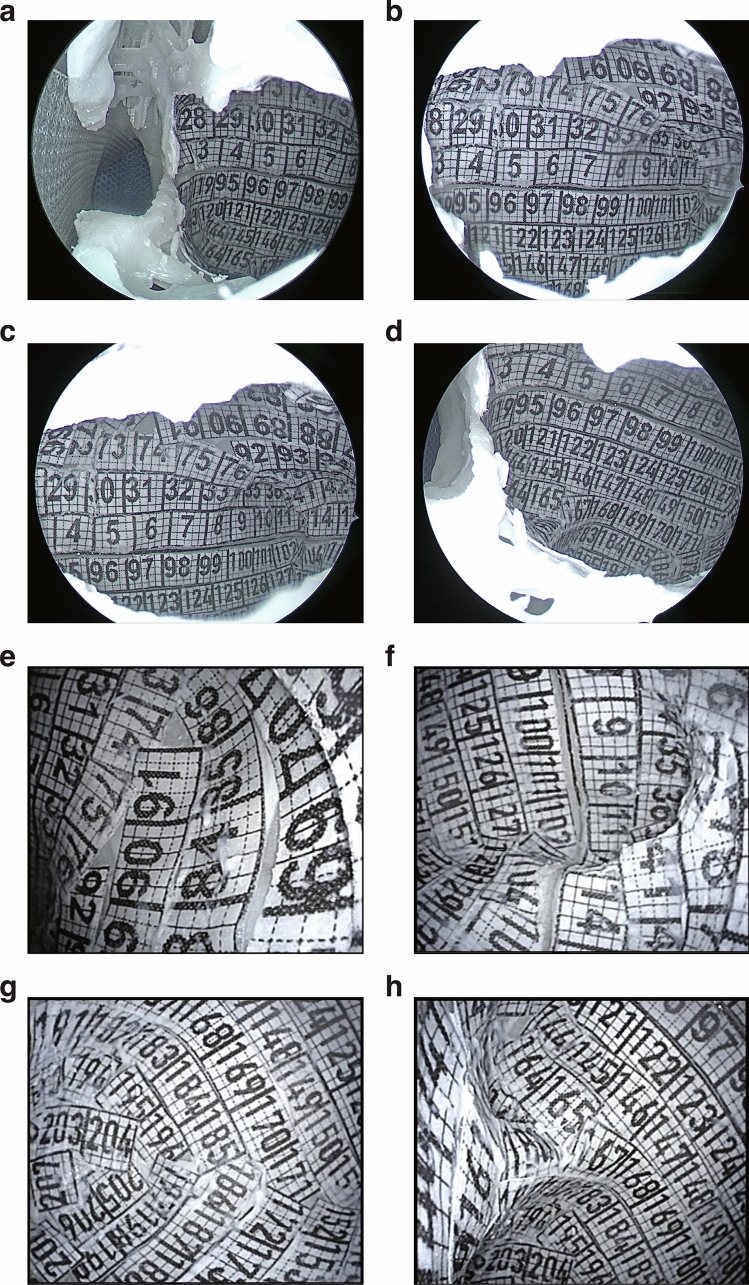
Representations of the inner surface in MS model observed by endoscopes. **a–d** The visual range gradually increased under 0° (**a**), 45° (**b**), and 70° (**c**, **d**) rigid endoscopes. **e–f** Flexible endoscope revealed superior wall (**e**), posterolateral wall, zygomatic recess and anterior wall (**f**), inferior wall (**g**), medial wall, inferior wall (**h**)

**Fig. 5:**
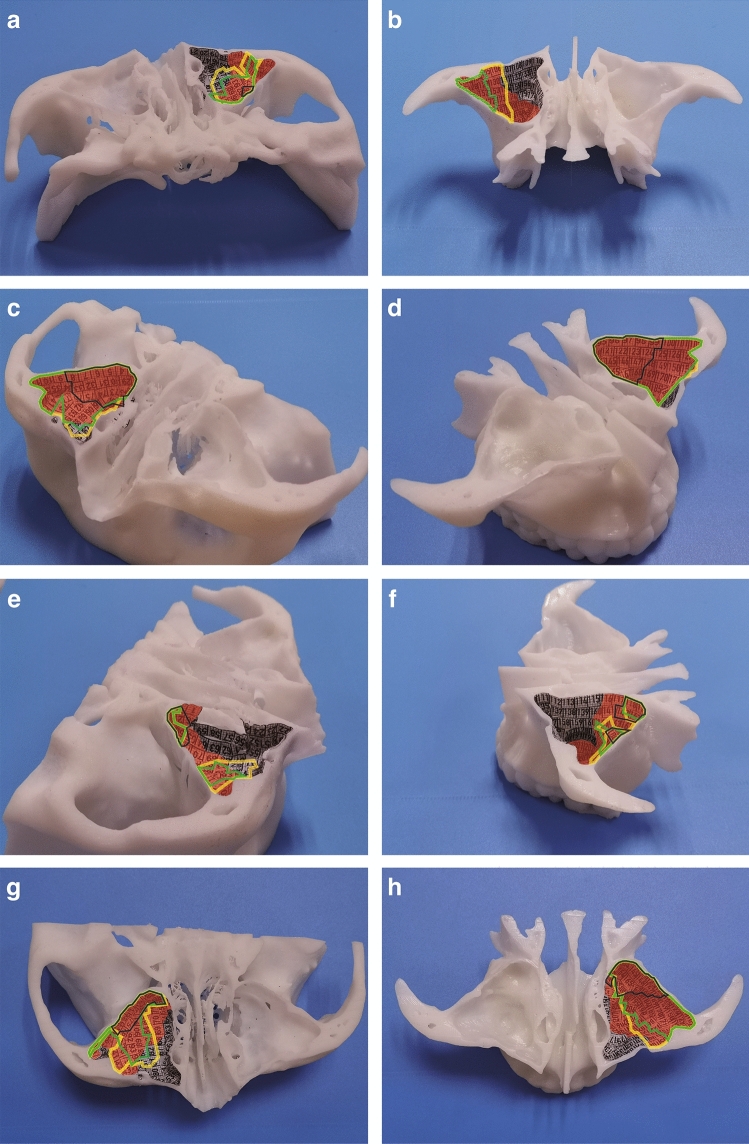
The visual range of MS model under rigid and flexible endoscopes. The visual range of 0°, 45°, 70° rigid endoscopes, and flexible endoscope were marked by black line, green line, yellow line, and red shadow, respectively. The view of anterior wall in the upper (**a**) and lower (**b**) parts, posterolateral wall of the upper (**c**) and lower (**d**) parts, medial wall of the upper (**e**) and lower (**f**) parts, superior wall (**g**), inferior wall (**h**) were observed by endoscopes (Color figure online)

Next, the two parts of the 3D-printed model are glued together. The walls with numbered grid papers are observed by different endoscopes, including 0° endoscope (Fig. 4a), 45° endoscope (Fig. 4b), 70° endoscope (Fig. 4c, d), and flexible endoscope (Fig. 4e–h). The observed numbers are recorded (Supplementary Table 2) and the visual ranges from different angles by different endoscopes were marked with different colors to be present dramatically (Fig. 5). We calculated the respective visible area and its percentage of each wall, as well as the total visible area and its percentage of total inner surface of MS under different endoscopes (Table 2).

**Table 2:** Statistics of visual range under rigid and flexible endoscopes

Visual range (Percentage)	Anterior wall	Posterolateral wall	Medial wall	Superior wall	Inferior wall	Total
0° endoscope	0 cm^2^ (0%)	7.625 cm^2^ (56.48%)	1.75 cm^2^ (13.46%)	0.875 cm^2^ (10%)	1.375 cm^2^ (23.91%)	11.625 cm^2^ (22.46%)
45° endoscope	1.875 cm^2^ (17.44%)	13.25 cm^2^ (98.15%)	1.5 cm^2^ (11.54%)	3 cm^2^ (34.29%)	2.625 cm^2^ (45.65%)	22.25 cm^2^ (43%)
70° endoscope	4 cm^2^ (37.21%)	13 cm^2^ (96.3%)	1.5 cm^2^ (11.54%)	4.5 cm^2^ (51.43%)	3 cm^2^ (52.17%)	26 cm^2^ (50.24%)
Flexible endoscope	5.5 cm^2^ (51.16%)	13.5 cm^2^ (100%)	4.125 cm^2^ (31.73%)	6 cm^2^ (68.57%)	5.125 cm^2^ (89.13%)	34.25 cm^2^ (66.18%)

The results revealed that the visual area under 0° endoscope, which is 11.625 cm^2^ (accounting for 22.46% of total area), is smallest, followed by an area of 22.25 cm^2^ (accounting for 43% of total area) under 45° endoscope (Table 2). More than half of the inner surface can be observed under 70° endoscope, with an area of 26 cm^2^ (accounting for 50.24% of total area). This visual range is largest among rigid endoscopes. The scope of observation by the flexible endoscope exceeds that of the rigid endoscopes, with an area of 34.25 cm^2^ (accounting for 66.18% of total area) (Table 2).

## Discussion

Inverted papilloma and malignant tumors originated frequently within the MS [30–32]. The extensive development of the techniques allows the effective treatment for lesions arising from each wall of the MS under endoscopy [6, 33]. Postoperative follow-up is essential for early detection of tumor recurrence, and further treatment. This could reduce the extent of surgery and prevent the metastasis for malignant tumors or malignant progress for inverted papilloma. However, it’s difficult to identify the recurrence in early stage through imaging tests, whether through MRI or CT. Endoscopy is still most intuitive and accurate in follow-up. However, the application value of rigid endoscope has great limitations, due to the limited visual range of the MS. At present, few studies have systematically explored the value of endoscopy for the postoperative follow-up of MS.

Our study investigated the visual range of MS under various endoscopes through image modeling, 3D printing technology, and physical measurement. Combined with clinical applications, we confirmed the value of flexible endoscopy in the postoperative follow-up of MS. In general, the visual range of the flexible endoscope is wider than that of the rigid endoscopes, while range of the 70° endoscope is largest among rigid endoscopes. Most walls of the MS can be observed, but there are still blind spots, including medial part of the anterior wall and the most part of medial wall.

The visual range of the posterolateral wall was widest among each wall of MS. More than a half range, located in the medial part of the wall, could be observed under 0° endoscope (Fig. 5c, d, Table 2). Therefore, it is suitable to use 0° endoscope for the follow-up of the tumor arising from medial part of the posterolateral wall, closed to the ostium of the MS. Moreover, almost all of the wall can be observed under all the other three types of endoscopes, including 45° endoscope, 70° endoscope and flexible endoscope (Fig. 5c, d, Table 2). Hence, these three types of endoscopes are appropriate in the postoperative follow-up for the tumor originating from the posterolateral wall of MS.

For the observation of the inferior wall, flexible endoscope has obvious advantages. It can display 89.13% area of the inferior wall, most of which concentrates around the medial part, almost completely covering the visible range under the 70° endoscope and other rigid endoscopes. In comparison, the visible range of the 0° endoscope is much smaller (Fig. 5h, Table 2). In case 2, tumors originated near the inferior wall where the rigid endoscope barely reach (Fig. 3m–o). By contrast, the entire lesion could be observed with a close-up view under flexible endoscope (Fig. 3s–u). Therefore, we recommend to use the flexible endoscope for postoperative follow-up when the primary tumor located on the inferior wall.

The visible range of the superior wall gradually increases in 0° endoscope, 45° endoscope, 70° endoscope and flexible endoscope. 0° endoscope can only display 10% of the superior wall, near the ostium of the sinus, where could be completely covered under 45° endoscope. 51.43% of the wall could be observed under 70° endoscope, overlapping the visible range of 45° endoscope. The flexible endoscope can reveal a wider range, accounting for 68.57% of the superior wall, most of which overlapped with the scope of 70° endoscope, with a slight difference (Fig. 5g, Table 2). Therefore, for the follow-up of tumors from superior wall of the MS, the flexible endoscope has the widest view, while 70° endoscope can be a supplement for some scopes.

The anterior wall is a blind spot under 0° endoscope. 45° endoscope can only display 17.44% area of the anterior wall, and that could be expanded to 37.21% by 70° endoscope. Flexible endoscope performed best, displaying 51.16% area of the anterior wall, which almost covering the observation range of rigid endoscopes. However, the medial part of the anterior wall still could not be visualized by any endoscope (Fig. 5a, b, Table 2). Although it was reported the feasibility of trans-oral access with flexible endoscope [34], the clinical application is difficult to achieve such a bending angle. More studies are needed to verify.

The visual range of the medial wall is largest under flexible endoscope, accounting for 31.73%. The range decreased under rigid endoscopes (Fig. 5e–f, Table 2). Therefore, the follow-up value of endoscopy for the medial wall is limited whether under rigid endoscopes or flexible endoscope. In clinical application, however, the tumor can be completely resected with the bone of the medial wall through the prelacrimal recess approach, which greatly reduced the probability of its in-situ recurrence.

Due to the variability of the MS in each person, the specific visual range in clinical application may vary from person to person. While the size of the ostium is distinctive for each person, even lesions were removed through the prelacrimal recess approach, the natural ostium should be expanded as much as possible during the operation to simplify the postoperative follow-up.

Although the operation of flexible endoscope is slightly more complex than that of 0° rigid endoscope, a well-trained otolaryngologist who is familiar with larynx examination using flexible endoscope is competent for the inspection of MS using flexible endoscope. Before operation, 0.05% oxymetazoline plus 1% tetracaine was used to help reduce nasal congestion and alleviate pain. The steps of operation are listed below. Operator holds the endoscope with left hand and assists the tip with right hand, then inserts the endoscope into the nostril gently and advances it slowly along the nasal cavity into the middle meatus. When operator reaches the plane of natural ostium of MS, bends the tip down with the the thumb controller and rotates the endoscope, orientating to the natural ostium before entering the MS.

We present some clinical experience in performing flexible endoscopy. Firstly, when the tip reaches the ostium of the MS, the neck of the flexible endoscope is controlled to bend down by the thumb controller. Since the "neck tip" part of the endoscope can only be bent upwards or downwards, it is necessary to rotate the endoscopic body about 90° with left hand at the same time. Correspondingly, the vision under the endoscope has also changed. This requires the operator to be skilled at the manipulation of the endoscope, as well as familiar with the structure and reference signs under the endoscope, so as not to “get lost” in nasal cavity and sinus. Secondly, when the flexible endoscope reaches the ostium of MS, it is usually recommended to start the neck bending at the posterior border of the ostium before entering, so as to obtain more room for rotation, enable smooth entry into the MS, and allow more freedom of movement inside the sinus.

The advancing direction of the flexible endoscope is the viewing direction, which makes it easy to understand. However, it is slightly more difficult to advance in the direction due to its flexibility. In contrast, the advancing direction is inconsistent with the viewing direction of the 45° or 70° rigid endoscope, which makes it confused, but the rigid endoscope is easier to control. In a word, the degree of difficulty for operation of them is similar.

In terms of comfort, all patients consistently have a better experience with the flexible endoscope and complain less discomfort during the operation. When examining the MS with a 45° or 70° rigid endoscope, the endoscope may often touch and squeeze the middle and inferior turbinates due to the difficulty in judging the advancing direction. In order to obtain largest visual range, the operator may constantly move the endoscope back and forth near the MS ostium to change the viewing angle, which will continuously increase the compression of the middle and inferior turbinates and cause discomfort to patients. The flexible endoscope is much softer to put less pressure on surrounding tissue. Therefore, for patients with narrow nasal cavity, pain sensitivity, or poor cooperation ability, the advantages of flexible endoscopy are more obvious.

Some flexible endoscopes also have an NBI module, which enables detection of small blood vessels under the mucosa [35]. It has been widely used in otolaryngology (mainly in pharynx and larynx) to identify premalignant and malignant lesions. In the examination of nasal cavity and sinus, the application value of NBI has been reported, preliminarily classifying the nasal and sinus mucosa under NBI [36–40]. That could be useful in diagnosing nasal diseases, especially in distinguishing benign and malignant tumors. In this study, the NBI was applied in one case to observe the characterization of lesion, with vascular dilation and aggregation (Fig. 3u, Supplementary Video). No typical vascular signs were observed, consistent with the benign postoperative pathology.

Another advantage of flexible endoscope is that some have an accessory channel through which matched biopsy forceps can pass. At present, biopsy technology under flexible endoscope is widely used in the larynx, hypopharynx, and gastrointestinal tract [41–43]. It can be tried in nasal sinuses. Forceps can be inserted for biopsy suspicious lesions, whose pathology results will help to guide the diagnosis and treatment. However, when biopsy forceps are inserted, the bending angle of the endoscope will be reduced to a certain extent. Therefore, development of flexible instruments suitable for MS will be a very attractive research direction.

In summary, flexible endoscope is dominant for displaying of each wall of the MS. We recommend flexible endoscopy as a routine practice in the postoperative follow-up for lesions within MS, especially from the anterior wall, medial wall, and inferior wall. If the lesion is located on the posterolateral wall, 45° endoscope and 70° endoscope are optional for follow-up, and 0° endoscope could be adequate for the lesion from the medial part of posterolateral wall. For the superior wall, although the visual range under 70° endoscope is slightly smaller than that of the flexible endoscope, 70° endoscope can be a supplement for some scopes.

## Supplementary Information

Below is the link to the electronic supplementary material.

Supplementary file1 (M4V 56555 kb)

Supplementary file2 (DOC 41 kb)

## References

[1] Sahlstrand-Johnson P, Jannert M, Strömbeck A, Abul-Kasim K (2011). Computed tomography measurements of different dimensions of maxillary and frontal sinuses. BMC Med Imaging.

[2] Cohen O, Warman M, Fried M, Shoffel-Havakuk H, Adi M, Halperin D, Lahav Y (2018). Volumetric analysis of the maxillary, sphenoid and frontal sinuses: a comparative computerized tomography based study. Auris Nasus Larynx.

[3] Whyte A, Boeddinghaus R (2019). The maxillary sinus: physiology, development and imaging anatomy. Dentomaxillofac Radiol.

[4] Lozano-Carrascal N, Salomó-Coll O, Gehrke SA, Calvo-Guirado JL, Hernández-Alfaro F, Gargallo-Albiol J (2017). Radiological evaluation of maxillary sinus anatomy: a cross-sectional study of 300 patients. Ann Anat.

[5] Palmer O, Moche JA, Matthews S (2012). Endoscopic surgery of the nose and paranasal sinus. Oral Maxillofac Surg Clin North Am.

[6] Suzuki M, Nakamura Y, Yokota M, Ozaki S, Murakami S (2017). Modified transnasal endoscopic medial maxillectomy through prelacrimal duct approach. Laryngoscope.

[7] Sathananthar S, Nagaonkar S, Paleri V, Le T, Robinson S, Wormald PJ (2005). Canine fossa puncture and clearance of the maxillary sinus for the severely diseased maxillary sinus. Laryngoscope.

[8] Zhou B, Han DM, Cui SJ, Huang Q, Wang CS (2013). Intranasal endoscopic prelacrimal recess approach to maxillary sinus. Chin Med J (Engl).

[9] Hildenbrand T, Weber R, Mertens J, Stuck BA, Hoch S, Giotakis E (2019). Surgery of inverted papilloma of the maxillary sinus via translacrimal approach-long-term outcome and literature review. J Clin Med.

[10] Lisan Q, Laccourreye O, Bonfils P (2016). Sinonasal inverted papilloma: from diagnosis to treatment. Eur Ann Otorhinolaryngol Head Neck Dis.

[11] Bugter O, Monserez DA, van Zijl F, Baatenburg de Jong RJ, Hardillo JA (2017). Surgical management of inverted papilloma; a single-center analysis of 247 patients with long follow-up. J Otolaryngol Head Neck Surg.

[12] Workman AD, Palmer JN, Adappa ND (2017). Posttreatment surveillance for sinonasal malignancy. Curr Opin Otolaryngol Head Neck Surg.

[13] Kim SA, Chung YS, Lee BJ (2019). Recurrence patterns of sinonasal cancers after a 5-year disease-free period. Laryngoscope.

[14] Martin J, Vayr F, Paris C, Vergez S, Krief P, Luc A, Corvisier J, de Gabory L, Herin F (2018). Nasal fibroscopy as a routine screening procedure of sinonasal adenocarcinoma of woodworkers: French longitudinal case study. Head Neck.

[15] Png LH, Heah HHW, Kang WL (2020). Use of flexible bronchoscopy in endoscopic sinus surgery for lateral frontal sinus mucoceles. Int Forum Allergy Rhinol.

[16] Tichenor WS, Adinoff A, Smart B, Hamilos DL (2008). Nasal and sinus endoscopy for medical management of resistant rhinosinusitis, including postsurgical patients. J Allergy Clin Immunol.

[17] Garcia J, Yang Z, Mongrain R, Leask RL, Lachapelle K (2018). 3D printing materials and their use in medical education: a review of current technology and trends for the future. BMJ Simul Technol Enhanc Learn.

[18] Wilk R, Likus W, Hudecki A, Syguła M, Różycka-Nechoritis A, Nechoritis K (2020). What would you like to print? Students' opinions on the use of 3D printing technology in medicine. PLoS ONE.

[19] Huang W, Zhang X (2014). 3D Printing: print the future of ophthalmology. Investig Ophthalmol Vis Sci.

[20] Bücking TM, Hill ER, Robertson JL, Maneas E, Plumb AA, Nikitichev DI (2017). From medical imaging data to 3D printed anatomical models. PLoS ONE.

[21] Chen YY, Lin KH, Huang HK, Chang H, Lee SC, Huang TW (2018). The beneficial application of preoperative 3D printing for surgical stabilization of rib fractures. PLoS ONE.

[22] Kwarcinski J, Boughton P, van Gelder J, Damodaran O, Doolan A, Ruys A (2021). Clinical evaluation of rapid 3D print-formed implants for surgical reconstruction of large cranial defects. ANZ J Surg.

[23] Birbara NS, Otton JM, Pather N (2019). 3D modelling and printing technology to produce patient-specific 3D models. Heart Lung Circ.

[24] Ten Kate J, Smit G, Breedveld P (2017). 3D-printed upper limb prostheses: a review. Disabil Rehabil Assist Technol.

[25] Louvrier A, Marty P, Barrabé A, Euvrard E, Chatelain B, Weber E, Meyer C (2017). How useful is 3D printing in maxillofacial surgery?. J Stomatol Oral Maxillofac Surg.

[26] Liang X, Liao W, Cai H, Jiang S, Chen S (2018). 3D-printed artificial teeth: accuracy and application in root canal therapy. J Biomed Nanotechnol.

[27] Liu P, Hu Z, Huang S, Wang P, Dong Y, Cheng P, Xu H, Tang B, Zhu J (2020). Application of 3D printed models of complex hypertrophic scars for preoperative evaluation and surgical planning. Front Bioeng Biotechnol.

[28] Crafts TD, Ellsperman SE, Wannemuehler TJ, Bellicchi TD, Shipchandler TZ, Mantravadi AV (2017). Three-dimensional printing and its applications in otorhinolaryngology-head and neck surgery. Otolaryngol Head Neck Surg.

[29] Valtonen O, Ormiskangas J, Kivekäs I, Rantanen V, Dean M, Poe D, Järnstedt J, Lekkala J, Saarenrinne P, Rautiainen M (2020). Three-dimensional printing of the nasal cavities for clinical experiments. Sci Rep.

[30] Jiang XD, Dong QZ, Li SL, Huang TQ, Zhang NK (2017). Endoscopic surgery of a sinonasal inverted papilloma: surgical strategy, follow-up, and recurrence rate. Am J Rhinol Allergy.

[31] Llorente JL, López F, Suárez C, Hermsen MA (2014). Sinonasal carcinoma: clinical, pathological, genetic and therapeutic advances. Nat Rev Clin Oncol.

[32] Mak W, Webb D, Al-Salihi S, Dadgostar A, Javer A (2018). Sinonasal inverted papilloma recurrence rates and evaluation of current staging systems. Rhinology.

[33] Zhou B, Huang Q, Sun J, Li X, Zhang W, Cui S, Shen PH, Wang C, Huang Z, Dong Y, Liang N (2018). Resection of inverted papilloma of the maxillary sinus via a prelacrimal recess approach: a multicenter retrospective analysis of surgical efficacy. Am J Rhinol Allergy.

[34] Trimarchi M, Tomazic PV, Bertazzoni G, Rathburn A, Bussi M, Stammberger H (2014). Video endoscopic oro-nasal visualisation of the anterior wall of maxillary sinus: a new technique. Acta Otorhinolaryngol Ital.

[35] Bertino G, Cacciola S, Fernandes WB, Fernandes CM, Occhini A, Tinelli C, Benazzo M (2015). Effectiveness of narrow band imaging in the detection of premalignant and malignant lesions of the larynx: validation of a new endoscopic clinical classification. Head Neck.

[36] Pagella F, Pusateri A, Chu F, Caputo M, Danesino C, Matti E (2013). Narrow-band imaging in the endoscopic evaluation of hereditary hemorrhagic telangiectasia patients. Laryngoscope.

[37] Torretta S, Gaffuri M, Cantarella G, Pignataro L (2013). Narrow-band imaging in the diagnosis of vascular nasal lesions. Am J Otolaryngol.

[38] Petersen KB, Kjaergaard T (2017). Role of narrow band imaging in the diagnostics of sinonasal pathology. BMJ Case Rep.

[39] Vlantis AC, Wong EWY, Ng SK, Chan JYK, Tong MCF (2019). Narrow band imaging endoscopy of the nasopharynx for malignancy: an inter- and intraobserver study. Laryngoscope.

[40] Bruno C, Fiori GM, Locatello LG, Cannavicci A, Gallo O, Maggiore G (2021). The role of narrow band imaging (NBI) in the diagnosis of sinonasal diseases. Rhinology.

[41] Pouw RE, Barret M, Biermann K, Bisschops R, Czakó L, Gecse KB, de Hertogh G, Hucl T, Iacucci M, Jansen M, Rutter M, Savarino E, Spaander MCW, Schmidt PT, Vieth M, Dinis-Ribeiro M, van Hooft JE (2021). Endoscopic tissue sampling—Part 1: upper gastrointestinal and hepatopancreatobiliary tracts. European Society of Gastrointestinal Endoscopy (ESGE) Guideline. Endoscopy.

[42] Pouw RE, Bisschops R, Gecse KB, de Hertogh G, Iacucci M, Rutter M, Barret M, Biermann K, Czakó L, Hucl T, Jansen M, Savarino E, Spaander MCW, Schmidt PT, Dinis-Ribeiro M, Vieth M, van Hooft JE (2021). Endoscopic tissue sampling—Part 2: lower gastrointestinal tract. European Society of Gastrointestinal Endoscopy (ESGE) Guideline. Endoscopy.

[43] Wellenstein DJ, de Witt JK, Schutte HW, Honings J, van den Hoogen FJA, Marres HAM, Takes RP, van den Broek GB (2017). Safety of flexible endoscopic biopsy of the pharynx and larynx under topical anesthesia. Eur Arch Otorhinolaryngol.

