# Supplementary material for: Flexible endoscopy in the visualization of 3D-printed maxillary sinus and clinical application
Source: Surg Endosc. 2022 Jul 26;36(10):7827–38. doi: 10.1007/s00464-022-09410-8 (PMC9485168; doi:10.1007/s00464-022-09410-8)
Supplement: Supplementary file 2 — Supplementary file2 (DOC 41 kb) [file 464_2022_9410_MOESM2_ESM.doc]

**Supplementary table 1** Numbers of grid papers corresponding to the walls of MS

| **The wall of MS** | **Numbers of grid papers** |
| --- | --- |
| **Anterior wall** | 12-21, 40, 45-48, 103-110, 128-134, 152-156, 172-175, 186-189 |
| **Posterolateral wall** | 3-11, 28-37, 73-76, 95-102, 120-127, 146-151, 167-171, 182-185 |
| **Medial wall** | 1-2, 22-27, 49-52, 54-62, 111-119, 135, 137-143, 157-163, 177-179, 181, 190-192 |
| **Superior wall** | 38-39, 41-44, 53, 63-72, 77-94 |
| **Inferior wall** | 136, 144-145, 164-166, 176, 180, 193-207 |

**Supplementary table 2** Numbers of grid papers under rigid and flexible endoscopes in each wall

| **The wall of MS** |  | **0° endoscope** | **45° endoscope** | **70° endoscope** | **Flexible endoscope** |
| --- | --- | --- | --- | --- | --- |
| **Anterior wall** | Complete | - | 14, 104, 128, 152, 172, 186 | 14, 104-106, 128-130, 152-153, 172-173, 186-187 | 12-16, 103-106, 128-130, 152-153, 172-174, 186-189 |
|  | Partial | - | 103, 105, 129 | 13, 15, 103, 131, 154, 188 | 17, 154 |
| **Posterolateral wall** | Complete | 3-8, 28-33, 73-75, 95-99, 120-124, 146-147, 167-168 | 3-11, 28-33, 35-36, 73-76, 95-102, 120-127, 146-151, 167-171, 182-185 | 3-11, 29-33, 36, 73-76, 95-102, 120-127, 146-151, 167-171, 182-185 | 3-11, 28-37, 73-76, 95-102, 120-127, 146-151, 167-171, 182-185 |
|  | Partial | 76, 148, 183 | 34, 37 | 28, 34-35, 37 | - |
| **Medial wall** | Complete | 27, 118-119,142-143 | 115, 118-119 | 115, 118-119, 143 | 1, 2, 27, 117-119, 141-143, 161-163, 181, 190 |
|  | Partial | 2, 26, 115-116 | 2, 116, 142-143, 161, 181 | 2, 142, 161, 181 | 26, 116, 140, 160, 179 |
| **Superior wall** | Complete | 66 | 41, 66, 72, 88-93 | 41-42, 66, 72, 82-83, 86-94 | 41-43, 65-66, 69-72, 77-79, 83-86, 89-93 |
|  | Partial | 65, 71, 72, 86, 91 | 42, 77, 78, 83, 86, 94 | 77, 78, 79, 81, 84, 85 | 39, 44, 64, 68, 88, 94 |
| **Inferior wall** | Complete | 144-145, 164-165, 180 | 144-145, 164-165, 180, 194-197, 204 | 144-145, 164-165, 180, 194-197, 204 | 144-145, 164-166, 180, 194-207 |
|  | Partial | 166 | 166 | 166, 198, 203, 205 | 193 |
